# Global Research Trends on Nanoplastics in Food: A Bibliometric Analysis of Human Health Concerns

**DOI:** 10.3390/foods14173102

**Published:** 2025-09-04

**Authors:** Suriyakala Gunasekaran, Sathiyaraj Sivaji, Kayeen Vadakkan, San Yoon Nwe, Sanith Sri Jayashan, Suchada Sukrong

**Affiliations:** 1 Department of Pharmacognosy and Pharmaceutical Botany, Center of Excellence in DNA Barcoding of Thai Medicinal Plants, Faculty of Pharmaceutical Sciences, Chulalongkorn University, Bangkok 103300, Thailand; suriyakala.g@chula.ac.th (S.G.); sanyoonnwe@gmail.com (S.Y.N.); sanith@tech.sab.ac.lk (S.S.J.); 2 School of Agro-Industry, Mae Fah Luang University, Chiang Rai 57100, Thailand; sathiyarajs92@gmail.com; 3 Amala Integrated Medical Research Department, Amala Institute of Medical Sciences, Thrissur 680555, Kerala, India; kayeenvadakkan@gmail.com; 4 Chulalongkorn School of Integrated Innovation, Chulalongkorn University, Bangkok 103300, Thailand

**Keywords:** bibliometric analysis, food, human health, nanoplastics, VOSviewer

## Abstract

The increasing prevalence of nanoplastics (NPs) in food and their potential implications for human health have become a growing concern in scientific and public health discourse. Using the Scopus database, this bibliometric analysis provides a comprehensive overview of global research trends on NPs in food from 2015 to 2024. Results show a significant increase in publications and citations post-2019. China is the top-ranked country in terms of the number of publications, citations, collaborations, affiliations, and funding sponsors. The most impactful documents were review articles, indicating that this research field is currently in a synthesis stage. The most productive source was *Science of the Total Environment*, with 21 articles, while 9 of the top 10 most productive journals were published by Elsevier, highlighting the field’s concentration in high-impact outlets. The most prolific authors were Wang, J., and Li, Y; Li, Y. was also the author with the most citation influence, with a h-index of 9. Keyword co-occurrence analysis showed seven thematic clusters formed from 50 individual keywords; the dominant terms were microplastics, NPs, and human health. These findings illustrate an evolving and interdisciplinary research field centered on evaluating the risks and detection of NPs in food and their implications for public health.

## 1. Introduction

Plastics have become a part of our lives in the 21st century, with products involving plastics playing an essential role in delivering the food we consume and in things that we use every day [1]. Plastics stay tied to various commercial matters as they are cheap, easy to produce, versatile, and hydrophobic. Global plastic production began around the 1950s and later increased significantly due to its widespread use in daily life [2]. Since then, global plastic production has increased from 1.5 million tonnes per annum to 374.8 million tonnes in 2019. A brief dip in production was observed in 2020 (375.5 million tonnes) due to disruptions caused by the COVID-19 pandemic, followed by further increases in 2022 (390.7 million tonnes) [3]. The trend of increasing global plastic production is expected to continue further, with global production levels of plastics projected to reach in the region of 940 million tonnes by 2040 [4]. A report was made based on the worldwide literature, which indicated that 79% of plastics have not been disposed of properly, resulting in significant plastic pollution [5,6]. Therefore, plastic pollution has become an international issue because of its impacts on human health, terrestrial, marine, and atmospheric environments.

Plastic pollution is not a new research topic. The study of plastic pollution began in the early 1960s when reports of marine species ingesting or becoming tangled in plastic pollution began to appear [1]. Then, in the 1970s, researchers documented plastic waste in the North Atlantic Ocean, further highlighting the growing concern regarding plastic pollution in marine environments [7]. In 2004, Thompson et al. [8] used the term “microplastics” (MPs) to describe the microscopic plastic debris seen in oceans, which was followed by a commonly accepted definition of MPs classified as plastic pollution less than 5 mm in at least one dimension [9]. Following this classification, nomenclature based on size discrimination was also applied, with terminology referring to mega-, macro-, meso-, micro-, and nano-plastics (NPs) [10].

NPs, ranging in size from 1 to 1000 nm, constitute another newly identified class of plastic contaminants derived from the breakdown of larger plastic wastes in the environment or intentionally manufactured as plastic microsystems for various applications [11]. These tiny particles have behaviors and properties that set them apart from MPs in that they may behave differently because they are considerably smaller, more reactive, and more mobile, with a greater surface-to-mass ratio, therefore making their behavior distinctly different when entering environmental or biological systems [12,13]. Because of their tiny size and high surface area, they have a greater potential to pass biological barriers or interfere with living systems and then carry harmful contaminants into biological systems [14]. They are also a cause for concern due to increased environmental and human health risks when increased reactivity, mobility, and potential for bioaccumulation and biomagnification in food webs are considered [15]. Even with the increasing prevalence and potential environmental and human health risks that NPs present, we still do not know much or have a general awareness of NPs, which calls for more research into their influences and mechanisms of action.

In the past 10 years, the scientific community has collectively turned its focus toward the study of NPs in food for two main reasons, namely their direct impact on human health and their recent detection in food such as bottled water, dairy, table salt, seafood, and crops. Therefore, chronic exposure through food consumption warrants further examination, as ingestion is the primary route by which NPs enter the human body [16]. The ingested NPs can traverse the gastrointestinal tract, potentially become bioavailable, and possibly induce cytotoxicity, oxidative stress, and immune response dysregulation or lead to changes in the gut microbiome [17]. NPs may also contain adsorbed contaminants such as persistent organic pollutants, heavy metals, and pathogenic microorganisms, further increasing its toxicological potential [18]. Hence, humans’ chronic exposure to NPs through food consumption has become an interesting area for scientific research. The field of study on NPs has included many disciplines to address this issue, such as environmental science, toxicology, food science, analytical chemistry, and public health. As a result, there is an urgent need to address the present research landscape of NPs in food to show trends, collaborations within research areas of focus, and remaining knowledge gaps.

Bibliometric analysis provides a structured and quantitative approach to reason about this need. By examining metadata from scientific publications, including publication counts, citations, authors and institutions, keywords, and collaboration networks, bibliometrics provides a view of research activity in a field [19]. This research activity includes access to impactful papers, active researchers, countries, and institutions, as well as monitoring thematic or topic evolution over time [20]. This study provides a bibliometric analysis of global research activity on NPs in food and their impact on human health. Earlier bibliometric studies focused broadly on MPs and combined micro- and nanoplastics (MNPs) [21,22,23,24]. In contrast, the current study focuses specifically on NPs, human health, and how they are treated scientifically in the literature.

The current study has multiple aims in creating a global research trend around NPs in food and their impact on human health. The first aim is to examine the number of scientific publications and the growth trend in this area of research to get a sense of the changes in scholarly inclination over time. Second, the study focuses on the leading contributors to global research in this area, which includes leading authors, countries, institutions, funding sponsors, and journals. It also provides an overview of the geography and institutions contributing to and engaging in research. Third, the study aims to find the patterns of collaboration and co-authorship trends to show the nature and size of research networks and linkages in the broader scientific community. Another key aim is to find the most cited and influential publications. Articles mentioned as influential could help serve as reference points for future studies. Additionally, the study helps to find emergent themes in research and the keywords used to highlight conceptual development and shifting areas of focus within the field.

## 2. Materials and Methods

### 2.1. Data Source and Search Strategy

Access to diverse scholarly databases and search engines, including Scopus, WoS, Science Direct, and Google Scholar, makes locating and retrieving scientific publications for bibliometric analysis significantly easier [25]. The data was collected using the Scopus database on 15 May 2025. Scopus is one of the leading and most comprehensive abstracting and citation databases of peer-reviewed literature [26]. A structured direct search query was used to ensure relevant, high-quality publications, focusing on the presence of NPs in food and their impact on human health.

We used the following search string in the TITLE-ABS-KEY field to capture relevant documents:

(TITLE-ABS-KEY (“nanoplastic*” OR “nano-plastic*” OR nano plastic*) AND TITLE-ABS-KEY (“food”) AND TITLE-ABS-KEY (“human health”)) AND PUBYEAR > 2014 AND PUBYEAR < 2025 AND (LIMIT-TO (DOCTYPE, “re”) OR LIMIT-TO (DOCTYPE, “ar”) OR LIMIT-TO (DOCTYPE, “ch”) OR LIMIT-TO (DOCTYPE, “bk”)) AND (LIMIT-TO (LANGUAGE, “English”)).

This search string was developed from a previous publication to retrieve documents directly linked to NPs, food, and human health in the title, abstract, or keywords [22]. The asterisk (*) in the search string acted as a wildcard operator, searching to include all words that preceded it. The search was limited to documents published between 2015 and 2024 to capture a recent research trend. To help ensure relevant and scholarly data, the search was also limited to English and the following document types: research articles, review articles, book chapters, and books. To assure the quality and reliability of our study, we focused on selected document types that are fully developed and peer-reviewed research outputs.

### 2.2. Data Export and Preparation

The retrieved bibliographic data was downloaded from Scopus in a CSV format with all the relevant metadata (i.e., authors, affiliation, titles, abstracts, keywords, years published, sources, and citations). Duplicate entries, irrelevant publications, and non-English records were manually inspected and removed.

### 2.3. Data Analysis

After gathering the data, we imported the search results into the Bibliometrics 4.5.0 R package and VOSviewer 1.6.20. These two programs allow more complex bibliometric analyses than other major software programs [27]. The Bibliometrics R software package is an open-source program that facilitates quantitative research in scientometrics and bibliometrics. It is developed in the R language, an open-source environment, and the software core quality comprises statistical algorithms, access to high-quality numerical routines, and the provision of integrated data visualization tools [28]. With R, we can perform various analyses, including publication trend analysis, author collaboration analysis, and keyword analysis, to comprehensively understand the research landscape. In addition, VOSviewer is a widely employed tool for visualizing and exploring bibliometric networks. It facilitates the identification of research clusters, the visualization of co-authorship networks, and the mapping of keyword co-occurrence networks [29,30].

## 3. Results and Discussion

### 3.1. Main Information About the Bibliometric Dataset

**Figure 1:**
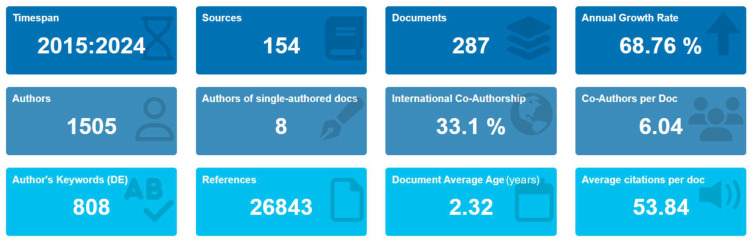
The main information of the refined documents.

The bibliometric dataset (Figure 1) used for this study covers the period from 2015 to 2024 and includes 287 documents published in 154 diverse sources. The dataset shows an extreme average growth rate of 68.76% per year, consistent with the growing number of researchers studying NPs in food and their effects on human health. The average age of documents is 2.32 years, which shows that most of the papers in this area are recent. The documents have a total of 26,843 references, and with an average of 53.84 citations per document, the dataset shows a significant amount of academic use. In terms of keywords, 808 author keywords and 2995 Keywords Plus provide a helpful perspective on the most often expressed themes and emphases in the literature. There are 1505 authors, and with only eight documents being single-authored, the collaborative nature of this research area is explicit. The average number of co-authors per document is 6.04, and 33.1% of the publications detail international collaborations, highlighting the global breadth of the research community around this issue.

Furthermore, for publication type, most documents are classified as review articles (137) and research articles (104); there are 45 book chapters and one book. Review articles in this discipline form a considerable part of the available literature. This only shows that the scientific community continues to combine existing knowledge and document any critical gaps in knowledge. Although the volume of articles continues to grow, original research and interdisciplinary research are necessary to resolve various outstanding questions related to exposure pathways, long-term health effects, and regulatory activities. It is important to conduct more empirical studies that link real exposure scenarios to measurable health outcomes. Building up this area of research will help facilitate linking studies of NPs with human risk assessments, thereby ensuring that scientific investigations are aligned with actual public health issues.

### 3.2. Subject Area Distribution

**Figure 2:**
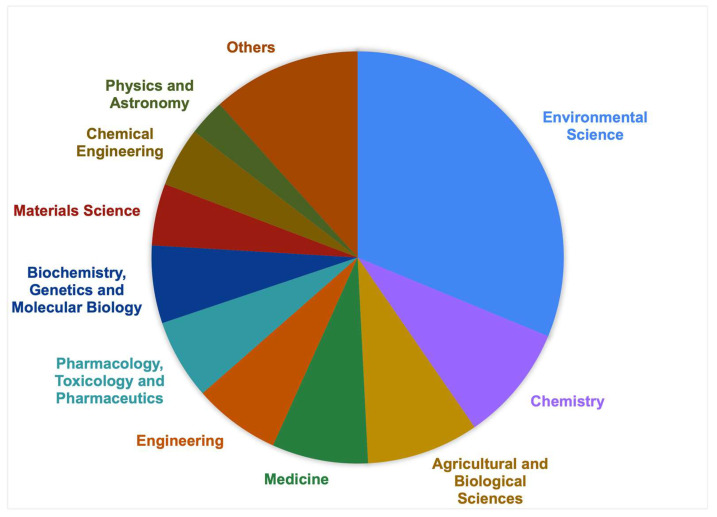
Distribution of publications based on their subject area.

The documents obtained for this bibliometric analysis were drawn from a broad range of subject areas, demonstrating the interdisciplinary nature of research on NPs in food and their impact on human health. A total of 287 documents were found covering many subject areas (Figure 2), with Environmental Sciences being the largest contributor (174 documents), followed by Chemistry (51), Agricultural and Biological Sciences (49), and Medicine (42). Engineering (38), Pharmacology, Toxicology, and Pharmaceutics (35), and Biochemistry, Genetics, and Molecular Biology (34) also made a substantial contribution, while smaller inputs from the Social Sciences, Computer Science, and Health Professions indicate interdisciplinary engagement.

The strong representation of Environmental Sciences confirms this area’s importance in understanding the distribution, degradation, and ecological hazards by the existing NPs in the environment. Chemistry shows the significance of analytical methods, molecular-level analysis, and testing for detecting and characterizing NPs from complex matrices such as food and water. The Agricultural and Biological Sciences and Medicine representations indicate a growing awareness of how NPs are transferred to food chains and the implications for human health. The need for toxicological and biochemical studies, further supported by Pharmacological and Molecular Biological studies, indicates the need to demonstrate cellular damage, oxidative stress, and the long-term effects of exposure. Engineering, Materials Science, and Chemical Engineering are making a growing effort to innovate and adopt detection tools, water filtration technologies, and safer and more sustainable alternatives to plastics. Social Sciences, Computer Science, and Business Management suggest that research moves beyond laboratories and recognizes the role of public awareness, data modeling, and regulatory policies in NPs. It is important to note that some publications are classified under more than one subject area, as interdisciplinary studies often span several domains. This diverse subject representation reinforces the complexity of the NP issue and the collaborative efforts needed to understand and address its impact on the environment, food systems, and human health.

### 3.3. Annual Publications and Citations Trend

**Figure 3:**
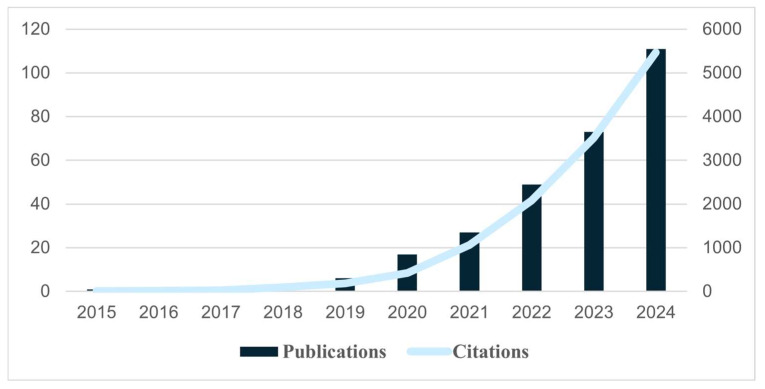
Annual trends in the number of publications and citations for the last decade.

The analysis of annual publication and citation trends from 2015 to 2024 indicates consistent and notable growth in scientific interest in NPs in food and their impact on human health (Figure 3). During the earliest phase (2015–2018), research activity was limited. Only one publication occurred in 2015 and 2016, no publications in 2017, and two in 2018. Regarding citations, they were also relatively low, with 10 in 2015, 12 in 2016, 31 in 2017, and 94 in 2018. The field began to grow from 2019 onwards. The number of publications grew to 6 in 2019, 17 in 2020, and 27 in 2021. Citation counts followed a similar pattern, with 189 in 2019, 422 in 2020, and 1064 in 2021. The finding represents a growing academic interest in the subject matter. The most substantial increase in activity occurred between 2022 and 2024. In 2022, 49 papers were published, 73 in 2023, and 111 in 2024. The citation of the documents increases dramatically for each of those years: 2079 in 2022, 3510 in 2023, and 5470 in 2024.

The annual publication and citation trends illustrate the rapid progression of NP research from refinement to an interdisciplinary area of study. In the early years (2015–2018), the few publications and citations indicate a lack of awareness, detection technology, and regulatory or public concern about NPs. The lack of publications in 2017 and 31 citations indicates early reliance on a few early studies that gathered academic attention. From 2019 to 2021, the field moved to a phase of growth. There were improvements in the chemical analytical methods to detect NPs, some initial evidence of the toxicological effects of NPs, and an increase in public recognition of the issue [26,31]. The last period, from 2022 to 2024, represents a fast-expanding field of growth. The number of published papers and citations indicates the level of adoption the field has globally, and the urgency researchers have as they tackle the non-trivial problem of NPs.

### 3.4. Country Analysis

**Figure 4:**
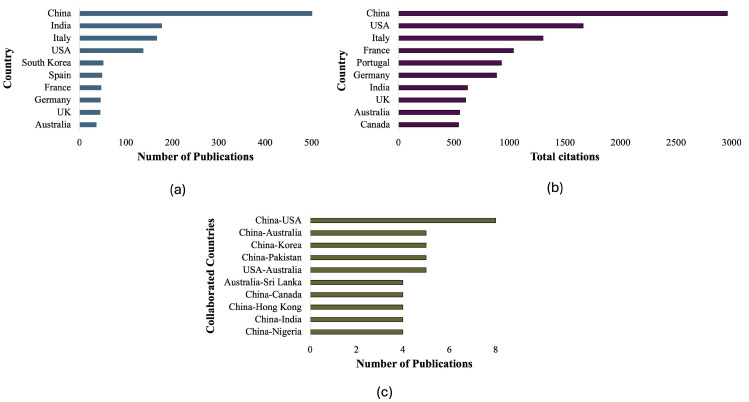
Country analysis is based on the number of publications (**a**), citation count (**b**), and collaboration with other countries (**c**).

Researchers from a total of 62 countries produced a total of 287 publications on NPs in food and human health. The top contributing countries, based on all the authors by publication volume (Figure 4a), were China (509), India (177), Italy (166), the USA (137), South Korea (51), Spain (48), France (46), Germany (45), United Kingdom (44), and Australia (36). Citation analysis of countries (Figure 4b) revealed that China’s total citations were 2961. Other top contributors were the USA (1663), Italy (1302), France (1036), Portugal (928), Germany (883), India (623), the United Kingdom (606), Australia (552), and Canada (542). Country collaboration analysis (Figure 4c) revealed that China had the highest international collaboration with multiple countries. The China–USA collaboration was the strongest, with eight joint publications. China also collaborated often with Australia, Korea, and Pakistan (five each) and had four collaborative publications each with Canada, Hong Kong, India, and Nigeria. Other noteworthy links included the USA–Australia collaboration (five joint publications) and the Australia–Sri Lanka partnership (four joint publications).

The results indicate that in terms of quantity and impact, China serves a significant role in the global advancement of NP-related research. China is noted to have many publications, citations, and international collaborations, which suggests that it has a strategic aim in NP-related research. The connections between China and the USA appear strong and possibly field-based, highlighting an effective and established partnership surrounding environmental health issues. Both India and Italy produced similar research on this topic; however, Italy’s impact was significantly higher than India’s. The USA had strong citations based on the amount of published research, which promotes the idea that some studies have a high-quality impact, while others are lower in quantity. European countries experienced moderate publications with high citation counts, indicating that although research may be focused or smaller in scale, it may have had a significant impact. Portugal is a high contributor, with lower publications producing many citations. China’s international collaboration with other developing nations and countries such as India, Nigeria, and Pakistan results in shared environmental concerns, international funding schemes, and bilateral scientific agreements. In addition, team collaborations, such as USA–Australia and Australia–Sri Lanka, indicate increasing involvement from the Asia–Pacific region and suggest an expanding global research network on NPs. The analysis shows that the current research agenda on NPs is collaboration-focused, geographically dispersed, and multifaceted, aimed at more effectively understanding and addressing multiple risks posed by NPs.

#### Co-Authorship by Country

**Figure 5:**
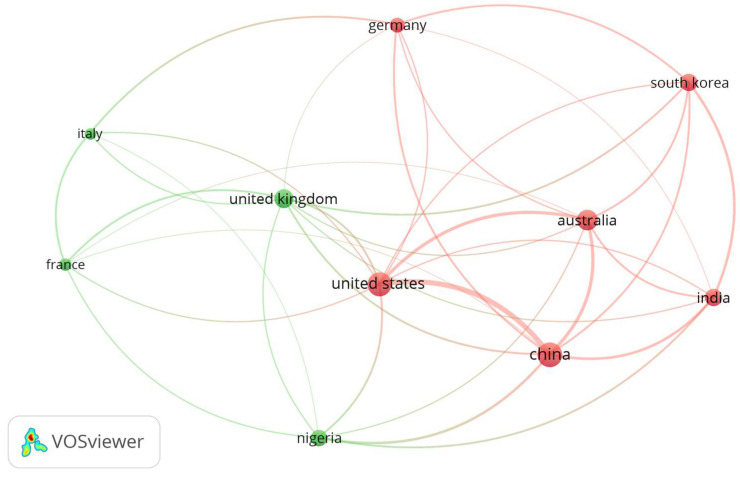
Co-authorship network visualization map of the top 10 countries.

The co-authorship analysis by country was conducted using full counting, with a minimum threshold applied for inclusion. Based on the data, the top ten countries with the highest levels of co-authorship activity by total link strength were identified and are given in Figure 5. China emerged as the most prolific contributor, with the highest total link strength (31), indicating its extensive international collaboration. The United States and the United Kingdom came in second and third, with total link strengths of 29 and 21, respectively. Other leading contributors included India, Italy, Germany, France, and South Korea, each with moderate link strengths ranging from 11 to 19. Australia and Nigeria, with fewer documents, stood out with high citation counts and moderate link strengths, reflecting impactful contributions and active participation in international collaborations.

### 3.5. Affiliation Analysis

The top 10 institutional contributions analysis revealed that the leading affiliations publishing on NPs in food and their impact on human health are from China and other major research countries (Table 1).

The predominance of Chinese institutions among the top contributors indicates that China is the leader in this research area in terms of publication volume and institutional capacity. The heavy output from a single institution, Guangdong Ocean University, and other institutions across China suggests that research hubs in many regions significantly contribute to research on NPs. The presence of institutions like Boston College and Rutgers University illustrates the level of participation by North America in this research area. Further, European institutions like Universitat Autònoma de Barcelona, the University of Milan, and the Joint Research Centre are significant contributors, showing widespread international interest and involvement in this research area. The institutional diversity suggests continued international engagement and recognition by research hubs in Asia, Europe, and North America and indicates that studying food and environmental pollution from NPs and measures to reduce the potential health impacts of NPs is essential. Recommendations for future collaborative efforts by the leading institutions could improve interdisciplinary research and collaboration, which can help accelerate knowledge and provide potential solutions to NP pollution.

### 3.6. Funding Sponsor

From the refined dataset, the studies published about NPs in food and human health received funding from 159 distinct funding agencies and 110 undefined funding agencies due to a lack of reporting. The undefined funding information limits the quality of the current research. This means that the analysis may underestimate the true level of funding support while potentially introducing bias to any conclusions surrounding how research activities are funded, including identifying the significant funding agencies. This key limitation highlights the need for funding sources to be transparent and standardized, allowing detailed mapping of funding patterns. Moreover, some studies were funded by more than one funding agency, suggesting cooperative funding or multi-source sponsorship. The top 10 funding agencies and their country are given in Table 2. The National Natural Science Foundation of China was the most noticeable funding sponsor and provided funding for 45 publications, the most significant number worldwide, followed by the Ministry of Science and Technology of the People’s Republic of China, which funded 35 studies. The European Commission funded 21 publications, and two articles were funded by Horizon 2020 and the Horizon 2020 Framework Programme. Others, including the National Research Foundation of Korea, funded twelve articles, and the Ministry of Science, ICT and Future Planning (Korea) funded eight articles, demonstrating a strong South Korean presence. UK Research and Innovation funded twelve publications, and the National Institutes of Health, USA, funded seven.

The funding pattern indicates that research investment has a geographical focus due to the predominance of Asian and European institutions in research funding, with China being the most influential country. The National Natural Science Foundation of China alone represented the most funded publications, highlighting China’s strategic focus on environmental toxicology and advanced materials research. Furthermore, the participation of multiple Chinese agencies demonstrates China’s efforts to become a leader in NP research. However, the vast number of publications with no funding information suggests that more reporting on funding sources would help identify the level of financial support for this area of research [32].

### 3.7. Document Analysis

In total, 287 documents on NPs in food and human health were collected and analyzed. Citation count identified the most cited documents. The top 10 highly cited documents had citations ranging from 453 to 885, signifying their significance in terms of scientific impact. The most cited document discussed the environmental and ecotoxicological risk of NPs [33], while other highly cited papers focused on human health analytics processes to detect NP and MP contamination in food items. Most of the documents are review articles reflecting the current stage of the field for synthesizing and evaluating emerging evidence. Few experimental studies have incorporated new developments in detecting MNPs from food matter.

The top 10 documents are summarized in Table 3 and discussed here. Da Costa et al. [33] reviewed the sources, environmental fate, and ecotoxicological consequences of NPs, emphasizing that their small size and large surface area enable them to penetrate higher trophic levels. Revel et al. [34] highlighted the human health implications of MNPs, which focus on the potential routes of exposure and two mechanisms of toxicity: (1) physical damage by the particles and (2) chemical toxicity from additives or adsorbed pollutants. Ivleva [35] highlighted the analytical challenges associated with detecting and quantifying MNPs. They made an excellent call for standardized methods of sampling, finding, and quantifying MNPs. Conti et al. [36] undertook one of the first empirical studies of MPs in fruits and vegetables and saw elevated contamination levels in apples and carrots. Rahman et al. [37] systematically analyzed over 17,000 publications. They found potential toxicological effects of MNPs, including a variety of areas for future research related to systematic gaps in knowledge, exposure pathways, and long-term effects. Shen et al. [38] aimed to characterize the toxicological effects of NPs in organisms and illuminated the role of particle size and surface chemistry in toxicity. In the review provided by Yuan et al. [39] on the definitions, characterization, and toxicology of MNPs in marine environments and food systems, they found human exposure pathways and described polymer-specific toxicity. Galloway [40] published a chapter with a collection of human health hazards caused by MNPs. Toussaint et al. [41] reviewed human dietary exposure to MNPs. Bradney et al. [42] reviewed particulate plastics as carriers of toxic trace elements in terrestrial and aquatic environments. The review explained how plastics absorb heavy metals and how this contamination impacts organisms and humans through exposure via food or air.

### 3.8. Most Relevant Journals

Research on NPs in food and human health has appeared in 154 sources. Table 4 displays the top 10 most productive journals, their number of publications, publishers, and journal quality. The leading journal is *Science of the Total Environment*, which has 21 articles published, the highest number, which makes it the principal source of NP-related research. The other productive journals are *Journal of Hazardous Materials* (17) and *Environmental Pollution* (10). Most significantly, it should be noted that 9 out of the 10 journals belong to Elsevier publishers, and 8 out of 10 sources are Tier 1 (T1) journals, reflecting high-impact, interdisciplinary research in NPs. The quality of the journals was assessed using the Scopus database available for the year 2024. T1 is the top 10th percentile of journal quality. Q1 and Q2 are the first and second quartile quality, marking the 25th and 50th percentile points. Moreover, of the 154 sources, 105 journals published one article, which shows that while the interest in NP-related research is wide-ranging and interdisciplinary, consistent publication is centered around a few leading journals.

### 3.9. Author Analysis

Of 1505 contributing authors, a small group represents the leading authors and contributors in the field of NPs research in food and human health (Table 5). Wang, J., was noted as the most prolific contributor, with 11 published articles, while Li, Y., followed with 10 publications. Other active contributors included Li, J., with six articles; Li, C., Li, X., Wang, B., Zhang, C., Zhang, X., and Zhang, Y., each with five articles; and He, L., with four publications, rounding out the top 10 contributors. In measuring scientific impact through the h-index, applicable to both productivity and citation influence, Li, Y. leads with a h-index of 9, meaning Li, Y. has received considerable academic recognition and consistent citation based on productivity. Wang, J. follows in h-index, with a h-index of 6, a reflective measure of productivity and quality of publication. Li, C., Li, J., Wang, B., and Zhang, X. each obtained a h-index of 5, and He, L., Kim, K.-H., Li, X., and Zhang, C. each had a h-index of 4. While many authors may have published multiple papers, only a few show firm productivity and citation impact, signifying their pivotal role in advancing the current understanding of NPs’ effects on food and human health.

#### Co-Authorship by Author Analysis

**Figure 6:**
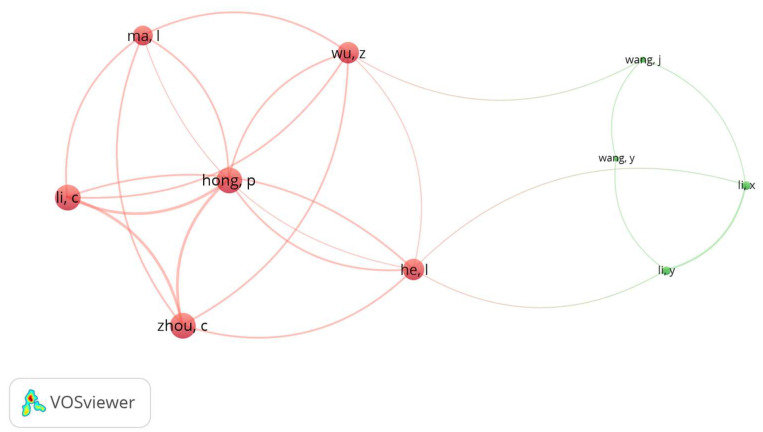
Co-authorship network visualization map of the top 10 authors.

Co-authorship by an author is an official indicator of the participation of two or more authors in a document. In a co-authorship network, nodes represent authors and are connected when they share the authorship of a document [43]. In this case, co-authorship by author analysis was undertaken to investigate the collaboration patterns between co-authors who published articles about NPs in food and associated human health outcomes. We applied a full counting approach, including a minimum threshold of three published documents and three citations per author. Out of 1361 authors, only 37 met this threshold. For each of the 37 authors, we found the total link strength to all co-authors to measure the intensity of the collaborations with those authors. For visualization purposes, we chose the top 10 authors with the greatest total link strength, as illustrated in Figure 6. The gap in network maps clearly shows two distinct clusters of collaboration, highlighting independent research groups with different thematic and/or institutional affiliations.

The results illustrate the structure and intensity of researchers’ collaborative networks in the field of NPs. The link strength is the intensity of an author’s collaborative ties with others [44]. Total link strength scores for collaborative ties are cumulative; thus, an author with a higher link strength is more heavily integrated into the scholarly network of researchers in this field and has contributed to and coordinated multi-author projects. Authors such as Hong, P., Li, C., and Zhou, C. showed the strongest collaborative links, each with a total link strength of 12. Their total link strength scores suggest that they play a prominent role in exchanging knowledge and/or producing research in collaboration with others. In a close-knit group or collaborative cluster, these authors contributed to common research aims and were very productive. Other authors of the note were He, L. and Wu, Z. (link strength: 10) and Ma, L (link strength: 9) based on this group’s research collaboration.

In contrast, the authors Li, X., Li, Y., Wang, J., and Wang, Y. registered lower link strengths (between two and four), meaning that their co-authoring interactions were less extensive. Notably, Li, Y. had the highest citation count (236) yet a lower link strength (4); this may show a more independent or limited form of co-authorship. This may be the case for Wang, J., who had the highest number of publications (11) of the listed authors, while also registering a low link strength (3), showing high productivity but low collaboration. The difference between citation impact and link strength demonstrates that extensive partnership does not always lead to impactful research [45]. On the other hand, high link strength will often also reflect sustained levels of co-authorship and the impact on the team.

### 3.10. Keyword Co-Occurrence Analysis

**Figure 7:**
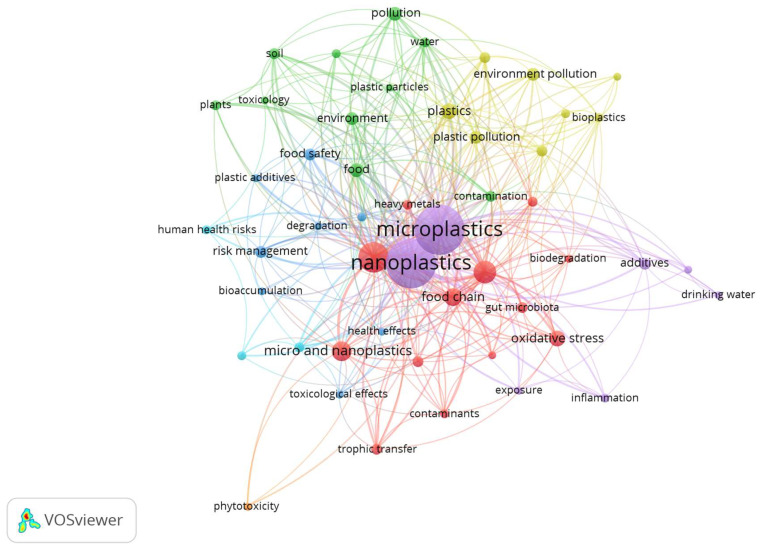
Top 50 NP research-related keyword co-occurrence visualization map.

Keyword co-occurrence analysis was conducted to find the leading themes in the studies on NPs in food and their effect on food. Overall, 736 keywords were extracted without any similar terms from the dataset. A minimum number of occurrences of three was used, resulting in 53 keywords. The top 50 keywords were included for network visualization (Figure 7). The top 10 most frequently occurring keywords in terms of occurrences were MPs (151 occurrences, 362 link strength), NPs (146, 329), human health (56, 165), toxicity (32, 97), food chain (20, 70), plastics (15, 52), pollution (12, 35), food (11, 38), plastic pollution (10, 34), and environment (10, 31). The top 50 keywords were represented in seven clusters with the full counting method: Cluster 1 (red, 13 keywords), Cluster 2 (green, 10 keywords), Cluster 3 (blue, 8 keywords), Cluster 4 (yellow, 8 keywords), Cluster 5 (purple, 7 keywords), Cluster 6 (light blue, 3 keywords), and Cluster 7 (orange, 1 keyword). Each keyword node size shows the frequency of its occurrence. The proximity of keywords shows the frequency with which keywords occur in titles, abstracts, or keyword lists.

#### 3.10.1. Cluster 1 (Red): Toxicity and Health Impacts

This cluster revolves around the toxicological and biological impacts of NPs, specifically on human health and ecological health. “Biodegradation, contaminants, cytotoxicity, oxidative stress, and toxicity” emphasizes the damage associated with NPs. The presence of NPs and the “food chain and trophic transfer” indicate NPs’ movement through the ecosystem, primarily the food web [46]. Keywords like “gut microbiota, heavy metals, and human health” reinforce the idea that exposure to NPs can be far-reaching, from altering the balance of microorganisms in the gut to accumulating toxins [47].

#### 3.10.2. Cluster 2 (Green): Environmental Contamination and Exposure

This cluster focuses primarily on environmental contamination and exposure pathways, especially where NPs are involved in terrestrial or aquatic environments. The main keywords include “contamination, environment, environmental health, food, plants, and pollution,” representing the enormous variety of contaminated ecological media. Keywords like “plastic particles, soil, toxicology, and water” suggest that NPs have entered the land and aquatic systems, raising potential health impacts on plants, animals, and humans [48]. The “environmental health” keyword suggests a human-centric view, connecting types of environmental pollution with risks to public health.

#### 3.10.3. Cluster 3 (Blue): Risk Assessment, Food Safety, and Regulatory Aspects of NPs

This cluster concerns regulatory and risk-related aspects and the public safety, particularly in dealing with plastic pollution. The keywords “bioaccumulation, degradation, emerging pollutants, food safety, and health effects” show concerns about the accumulation of plastics and related chemicals into food chains and the unknown or evolving risks of these pollutants. Risk management, toxicological effects, and plastic additives underscore the attention given to assessment and control mechanisms despite significant challenges to managing the human health risks of plastics and their products [49]. The cluster highlights the increasing interest in integrated safety in responding to emerging challenges that NPs produce.

#### 3.10.4. Cluster 4 (Yellow): Plastic Waste and Sustainable Alternatives

Cluster 4 focuses on plastic production and the environmental waste it creates. The terms “bioplastics, environment pollution, food packaging, health risks, plastic pollution, plastic waste, and plastics” denote a strong emergent concern with the entire lifecycle of plastic material to waste disposal and its contributions to environmental impact. The keyword “health impacts” suggests human dimensions of environmental pollution, while bioplastics capture sustainable alternatives.

#### 3.10.5. Cluster 5 (Purple) MNPs in Health Impact

This cluster is linked with human health and the risk of direct exposure to MNPs and their additives. Keywords in this cluster include “additives, drinking water, exposure, health impacts, inflammation, MPs, and NPs,” which represent avenues of entry into the human body (e.g., ingestion and inhalation) [50]. The mention of “inflammation” implies biological responses that can start with plastic particulates or chemical residues [51]. MNPs have been associated with respiratory conditions, including chronic inflammation, asthma, pulmonary fibrosis, and possibly lung cancer. Ingested MNPs can affect the gastrointestinal tract and microbiome, and have been associated with inflammatory bowel disease, irritable bowel syndrome, autoimmune diseases, and nutrient malabsorption [52,53,54,55]. While less is known about the impact of MPs and NPs on the integumentary system, they have been reported to disrupt skin integrity, cause local inflammation, and influence skin homeostasis [56]. This cluster represents increasing concern about how NPs might interact with human physiology in chronic health conditions. It shows the urgent need for toxicological studies and risk reduction methods to deal with this challenge of significant concern.

#### 3.10.6. Cluster 6 (Light Blue): Internalization of NPS

This small but critical cluster focuses on specific health risk endpoints and specific exposure pathways. This cluster includes “human exposure, human health risks, and translocation,” representing the mechanisms of movement of NPs in the human body and their potential to cross biological barriers [57]. Leslie et al. [58] reported that of the blood samples collected from healthy volunteers in the Netherlands, 77% contained NPs, suggesting that they are able to cross epithelial barriers (e.g., gut, lung) and enter systemic circulation. In line with this, Marfella et al. [59] noted that MNPs, predominantly polyethylene and PVC, were embedded in the arterial plaque of 58% of asymptomatic patients undergoing carotid endarterectomy. The finding of plastic particles in vascular tissue is strong support of their affinity for translocation and potential accumulation in tissue.

#### 3.10.7. Cluster 7 (Orange): Phytotoxic Effects of NPS

This cluster contains just one keyword, “phytotoxicity,” which refers to the effect of NPs on plants. Although it only contains one keyword, it represents a significant and emerging concern in the literature about how plastic pollutants affect plants, including plant health, soil health, and agricultural productivity [60]. Smaller MNPs may also be more mobile in soil and allow for greater exposure to seed coats and possible effects on water and nutrients during germination than plastic debris in the form of macroparticles [61]. The type, concentration, and surface charge of an MNP type affect seed germination. High concentrations of MNPs can have phytotoxic effects, reducing seed germination and seedling vigor [62], while surface charge affects the availability of nutrients and water in the soil [63].

All clusters are connected through a flow that starts with plastic waste and sustainable alternatives (Cluster 4), which represent the sources and potential solutions to plastic pollution. This pollution leads to environmental contamination and exposure (Cluster 2), where NPs enter terrestrial and aquatic ecosystems. From there, NPs move through the food web, causing toxicity and health impacts (Cluster 1) on humans and wildlife. These health concerns prompt risk assessment, food safety, and regulatory efforts (Cluster 3) aimed at managing the risks posed by NPs. Detailed understanding of human exposure pathways and biological internalization (Clusters 5 and 6) informs the assessment of health risks by revealing how NPs enter and affect the body. Finally, phytotoxic effects (Cluster 7) show the impact of NPs on plant and soil health, connecting to environmental contamination and influencing food safety. Altogether, these clusters illustrate an integrated research framework covering the lifecycle of NPs, their environmental and health impacts, and the responses aimed at mitigation.

### 3.11. Thematic Map Analysis

**Figure 8:**
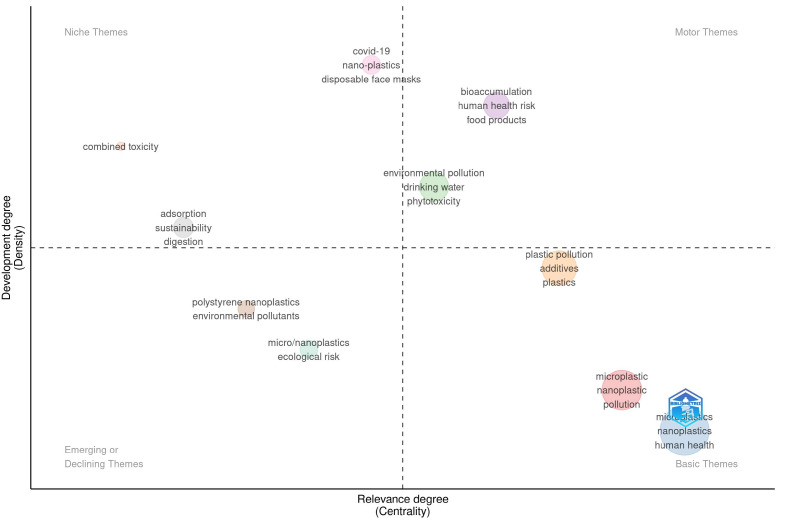
Thematic map visualization.

A thematic map was created using author keywords from the dataset to visualize the conceptual structure of NP-based research in food and human health contexts (Figure 8). The map has four quadrants, motor themes, niche themes, emerging or declining themes, and basic themes, determined according to two dimensions: centrality (degree of relevance) and density (degree of development) [64]. The basic theme quadrant (low density, high centrality) included “MPs, NPs, pollution, and human health”. This quadrant shows that plastic particles are common contaminants, and there is concern about potential impacts on human health. It provides a conceptual foundation for much of the research in the field. However, the underdevelopment of this theme is evidence of a lack of clinical, mechanistic, or long-term epidemiological studies linking exposure to NPs with a medical pathology in humans, such as inflammation, endocrine disruptors, genotoxicity, or carcinogenicity.

In contrast, the motor theme quadrant (high density and centrality) includes “bioaccumulation, human health risk, food products, environmental pollution, drinking water, and phytotoxicity,” which are well-developed and highly central to the field. The keywords “food products” and “human health” in motor themes raise concern about how the NPs enter the food chain and reach humans and the systemic effects of these particles once ingested. A recent study has indicated that NPs can translocate across biological membranes, enter systemic circulation, and elicit oxidative stress, immunotoxicity, or metabolic alterations [65]. This cluster strongly shows the shift in NPs from the environmental to the biomedical field.

The niche themes (high density, low centrality) are recently raised topics, which include “COVID-19, nano-plastics, disposable face masks, combined toxicity, adsorption, sustainability, and digestion”. The COVID-19 pandemic has amplified the concern of plastic-based personal protection equipment (PPE) waste entering aquatic and terrestrial systems, fragmenting into MNPs, and eventually re-entering human exposure routes [66]. These themes coherently overlap with combined toxicity, where NPs interact with other existing chemicals or heavy metals, which could increase the toxic effects. Through niche themes, they offer novel insights into real-world exposure contexts and complex toxicological interactions, essential for risk assessment.

The emerging or declining theme (low centrality and density) quadrant includes keywords such as “MNPs, ecological risk, environmental pollutants, and polystyrene NPs.” Each of these shows either essential but unresolved pressures or declining research areas. Ecological risk, as the name implies, is not directly linked to human health; however, if NPs disrupt key organisms that are part of the food web, they could negatively affect the ecosystem services that support food security and nutritional value. Polystyrene NPs are continuously utilized as model systems in NP toxicity studies. However, they are not what researchers necessarily mean in terms of potential food implications, and they are relevant to the discussion because they may ultimately degrade during the storage or heating of foods [67].

## 4. Conclusions

The current bibliometric analysis confirms rapid research growth and the interdisciplinary nature of research on NPs in food and human health. This field of study has received significant scientific interest over the past decade, further shown by the recent growth in publications and citations. The strength of review articles in this field shows that the scientific community is still trying to amalgamate existing knowledge and find gaps in knowledge and research opportunities. Most publications were from Environmental Sciences, Chemistry, and Biological Sciences. Still, publications from different subject areas, including Medicine, Pharmacology, Engineering, and various Social Sciences, show the field’s interdisciplinary nature. China and its respective National Natural Science Foundation and the European Commission are major funding organizations that have published the most and have the highest percentage of any nation’s collaboration. Keyword co-occurrence analysis revealed seven thematic clusters, most dominated by themes related to toxicity, environmental contamination, safety for food products, plastics waste, human exposure, and concerns with regulatory practices. While this provides a rich overview of existing knowledge, the lower incidence of experimental evidence producing studies, specifically those directly linking NPs exposure to human health outcomes, underscores the need for more targeted, in vivo, and longitudinal studies.

Although there have been some advancements in the literature, there are still many unknowns in this area. First, we still lack approved methods for finding and quantifying NPs in complex food matrices. Secondly, all toxicological studies are either in vitro or in animal models. There is a lack of data about the effects of chronic, low-dose exposure in humans. Third, many countries have no risk assessment schemes to regulate NPs in food and beverages. Addressing these gaps requires greater collaboration, including food technologists, toxicologists, environmental chemists, and medical researchers. Research efforts should focus on developing standardized detection protocols, thresholds for exposure, and longitudinal epidemiological studies. There is a need to create alternative plastics and implement guidelines to limit plastics in food packaging and processing. To conclude, the research field of NPs in food and their impact on human health is growing, reflecting pressures of urgent global significance. This study should serve as a stepping stone toward future scientific research, the development of public health, and the design of sustainable solutions to mitigate the impact of NPs on human health.

## Figures and Tables

**Table 1:** The most influential affiliation and its country.

Affiliation	Country	Articles
Guangdong Ocean University	China	37
Boston College	US	18
Universitat Autònoma De Barcelona	Spain	15
University Of Milan	Italy	15
Chongqing University	China	14
Joint Research Centre (JRC)	Belgium	14
Nanjing Normal University	China	13
Rutgers University	US	13
Sun Yat-Sen University	China	12
University Of Salerno	Italy	12

**Table 2:** Top 10 funding sponsors in the field of NP-related research.

Funding Sponsor	Country	Articles
National Natural Science Foundation of China	China	45
Ministry of Science and Technology of the People’s Republic of China	China	35
European Commission	Europe	21
National Research Foundation of Korea	Korea	12
UK Research and Innovation	UK	12
National Key Research and Development Program of China	China	10
Ministry of Science, ICT and Future Planning	Korea	8
Horizon 2020	Europe	7
Horizon 2020 Framework Programme	Europe	7
National Institutes of Health	US	7

**Table 3:** Top 10 most cited documents.

Paper	Document Type	Total Citations	Reference
Da Costa JP, 2016, Sci. Total Environ.	Review	885	[33]
Revel M, 2018, Curr. Opin. Environ. Sci. Health	Review	581	[34]
Ivleva NP, 2021, Chem. Rev.	Review	579	[35]
Oliveri Conti G, 2020, Environ. Res.	Research	563	[36]
Rahman A, 2021, Sci. Total Environ.	Research	541	[37]
Shen M, 2019, Environ. Pollut.	Review	526	[38]
Yuan Z, 2022, Sci. Total Environ.	Review	484	[39]
Galloway TS, 2015, Marine Anthropogenic Litter	Book Chapter	483	[40]
Toussaint B, 2019, Food Addit. Contam. Part A Chem. Anal Control Exposure Risk Assess	Review	470	[41]
Bradney L, 2019, Environ. Int.	Review	453	[42]

**Table 4:** Top 10 most productive journals.

Sources	Articles	Publisher	Quality of the Journal
Science of the total environment	21	Elsevier	T1
Journal of hazardous materials	17	Elsevier	T1
Environmental pollution	10	Elsevier	T1
Environmental research	9	Elsevier	T1
Chemosphere	6	Elsevier	T1
Environment International	5	Elsevier	T1
Heliyon	5	Elsevier	Q1
Toxics	5	MDPI	Q2
Trac–trends in analytical chemistry	5	Elsevier	T1
Nanoimpact	4	Elsevier	T1

**Table 5:** Top 10 most productive authors based on their number of publications and h-index.

Author Analysis by Publications		Author Analysis by h-Index	
Authors	Articles	Author	h-Index
Wang, J.	11	Li, Y.	9
Li, Y.	10	Wang, J.	6
Li, J.	6	Li, C.	5
Li, C.	5	Li, J.	5
Li, X.	5	Wang, B.	5
Wang, B.	5	Zhang, X.	5
Zhang, C.	5	He, L.	4
Zhang, X.	5	Kim, K.-H.	4
Zhang, Y.	5	Li, X.	4
He, L.	4	Zhang, C.	4

## Data Availability

The original contributions presented in the study are included in the article, further inquiries can be directed at the corresponding author.

## Abbreviations

The following abbreviations are used in this manuscript:

NPs	Nanoplastics
MPs	Microplastics
MNPs	Micro- and nanoplastics
T1	Tier 1
PPE	Personal protection equipment
